# Effects of Walking in a Forest on Young Women

**DOI:** 10.3390/ijerph16020229

**Published:** 2019-01-15

**Authors:** Chorong Song, Harumi Ikei, Takahide Kagawa, Yoshifumi Miyazaki

**Affiliations:** 1 Center for Environment, Health and Field Sciences, Chiba University, 6-2-1 Kashiwa-no-ha, Kashiwa, Chiba 277-0882, Japan; crsong1028@chiba-u.jp (C.S.); ikei0224@ffpri.affrc.go.jp (H.I.); 2 Forestry and Forest Products Research Institute,1 Matsunosato, Tsukuba, Ibaraki 305-8687, Japan; kagawa@ffpri.affrc.go.jp

**Keywords:** shinrin-yoku, forest therapy, brief walks, females, heart rate variability, blood pressure, pulse rate, semantic differential method, Profile of Mood State, State–Trait Anxiety Inventory

## Abstract

The effects of forest activities on health promotion have received increasing attention. The aim of this study was to evaluate the physiological and psychological effects of brief walks in forests on young women. The experiments were conducted in 6 forests (test) and 6 city areas (control). Overall, 12 participants in each area (60 participants in total, mean age: 21.0 ± 1.3 years) were instructed to walk in a forest and a city area for approximately 15 min; simultaneously, their heart rate variability, heart rate, blood pressure, and pulse rate were measured to quantify their physiological responses to walking. The modified semantic differential method, Profile of Mood States (POMS), and the State–Trait Anxiety Inventory (STAI) were used to determine their psychological responses. Walking in a forest was associated with significantly higher parasympathetic nervous activity and lower sympathetic nervous activity and heart rate. In addition, scores for the comfortable, relaxed, and natural parameters and vigor subscale of POMS were significantly higher, whereas scores for negative feelings, such as tension–anxiety, depression–dejection, anger–hostility, fatigue, and confusion, were significantly lower, as were the total mood disturbance of POMS and the anxiety dimension of the STAI. The subjective evaluations were generally in accordance with the physiological responses. A brief walk in a forest resulted in physiological and psychological relaxation effects in young women.

## 1. Introduction

People are exposed to many stressors in daily life; in response, they try to find effective methods to cope with stress and to relax. One result of this has been the increased focus on using forest environments as places for promoting health through taking in the forest atmosphere, an approach known as “shinrin-yoku” or “forest bathing” [1,2]. It has been suggested that forest bathing, which is based on proven effects of a forest environment such as inducing relaxation, can improve the health of the body and mind. The accumulation of data has resulted in the concept of “forest therapy”; this refers to evidence-based forest bathing with the aim of achieving a preventive medical effect by inducing physiological relaxation and immune system recovery [3]. 

Numerous studies have demonstrated the effects of forest environments in mitigating stress states and inducing physiological relaxation [4,5,6,7,8,9,10,11,12,13]. Studies on healthy young men have demonstrated that time spent in a forest environment, such as walking through a forest and/or viewing the scenery, can reduce levels of salivary cortisol, a stress hormone [4,5,6,7,8,9,10,11], blood pressure [5,8,9,12], and pulse rate [5,7,8,10,12], as well as increase parasympathetic nervous activity, which is enhanced in relaxing situations [5,7,9,10,11,12,13]. In addition, sympathetic nervous activity, which increases in stressful situations, is suppressed [5,9,10,11,12,13], and there is a decrease in the cerebral blood flow in the prefrontal cortex [6]. A forest therapy trip has also been shown to increase natural killer (NK) cell activity and improve immunity, with these effects lasting for approximately one month [14,15]. 

In addition to many studies targeting healthy young men, studies including large sample sizes [9,16] and population-based studies, have also been reported [17,18,19]. In a study involving 420 participants, Park et al. [16] demonstrated that forest therapy mitigated stress and led to physiological relaxation, as evidenced by the index of heart rate variability (HRV), blood pressure, pulse rate, and salivary cortisol concentration. They performed the experiments at 35 locations throughout Japan including 12 participants in each area and summarized the data. Kobayashi et al. evaluated the characteristics of HRV distribution for 625 participants who viewed forest scenery [17] and 485 participants who walked in forests [19] as well as the characteristics of salivary cortisol concentration for 348 participants who viewed forest scenery [18] by collecting data from 12 participants in each area. In addition, recent studies involving elderly individuals and adults at risk of stress- and lifestyle-related diseases, such as high blood pressure, diabetes, and depression, have demonstrated positive physiological effects resulting from various activities in forests [20,21,22,23,24,25,26,27,28].

However, most studies involving forest therapy experiments have reported the various effects on male subjects [4,5,6,7,8,9,10,11,12,13,14,15,16,17,18,19,24,25,28], with few reports focusing on female subjects [23,29,30,31]. In a study of elderly women, Lee and Lee [23] reported that walking in a forest for 1 h improved arterial stiffness and pulmonary function. Ochiai et al. [29] investigated the effects of a one-day forest therapy program on middle-aged women and reported that the pulse rate and salivary cortisol levels decreased significantly following the program. Li et al. [30] examined the effects of a three-day, two-night trip to the forest on young women and reported that NK cell activity increased and remained at a raised level for more than 7 days after the trip. Kim et al. [31] also reported enhanced NK cell activation; their study involved patients with breast cancer who underwent daily forest therapy for 14 days while living in an accommodation in the forest. The increased NK cell activity led to the production of two anticancer molecules [31]. However, these studies were limited by their small sample sizes. Further data are required.

The aim of the present study was to clarify the physiological and psychological effects on young women of brief walks through forests. 

## 2. Materials and Methods 

### 2.1. Participants 

Between 2014 and 2017, we conducted experiments in six forests and six city areas in Japan. In each region, we selected safe, well-maintained forest areas, as well as city areas that were located either downtown or near a Japan Railway station. Twelve Japanese female university students participated in each experiment (a total of N = 72 participants). None of the participants reported a history of physical or psychiatric disorders. Of the 72 participants, data from 60 participants (mean age, 21.0 ± 1.3 years) were analyzed, because of errors in the data collection (Table 1). During the study period, the participants were prohibited from consuming alcohol and tobacco, and caffeine consumption was controlled. The study was approved by the Ethics Committee of the Center for Environment, Health, and Field Sciences, Chiba University, Japan (project identification code number: 5). Before starting the experiments, we explained the aims and procedures of the study to the participants and obtained their written informed consent.

### 2.2. Experimental Design

To eliminate order effects, the 12 participants were randomly divided into two groups of six. One group performed the experiment in the forest area, and the other performed the same experiment in the urban area. On the following day, the groups switched field sites. The details regarding the experiment sites in the forest have been summarized in Table 2.

**Figure 1:**
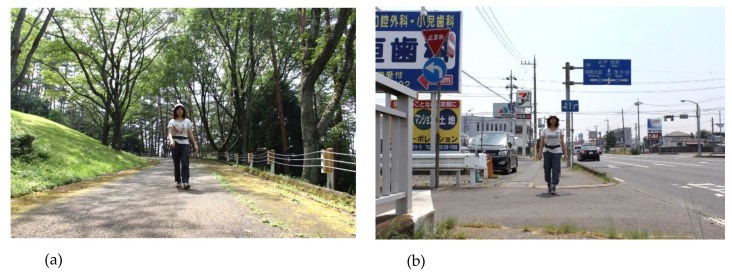
Walking through forest (**a**) and city (**b**) areas.

On arrival in the forest or city area, the participants waited in a waiting room and were taken one at a time to the experimental site. After resting in a chair for 5 min at the site, the participant’s blood pressure and pulse rate were measured to obtain the values before walking. The participant then walked along a given course in the experimental area for approximately 15 min at her normal walking pace (Figure 1). The walking course in each area was approximately 1 km, and the distances between the forest and city areas were almost same in each area. During the walking period, her HRV and heart rate were measured continuously. After completing the walk, the participant rested for 5 min in a chair and her blood pressure and pulse rate were again measured. After completing all the physiological measurements, the participant underwent the subjective assessment and returned to the waiting room.

### 2.3. Physiological Measurements

The physiological measurements made in this study were of HRV, heart rate, blood pressure, and pulse rate. HRV was analyzed for the periods between consecutive R waves (R–R intervals), measured by a portable electrocardiograph (Activtracer AC-301A, GMS, Tokyo, Japan) [32,33]. This device used a three-lead electrocardiogram (Lead II) to obtain the necessary measurements. Power levels for the high-frequency (HF, 0.15–0.40 Hz) and low-frequency (LF, 0.04–0.15 Hz) components of HRV were calculated using the maximum entropy method (MemCalc/Win, GMS, Tokyo, Japan) [34,35]. The HF power is considered to be an indicator of parasympathetic nervous activity and the LF/HF power ratio an indicator of sympathetic nervous activity [36,37]. To normalize the HRV parameters across the participants, the HF power and LF/HF ratio values were transformed to their natural logarithmic values (ln(HF) and ln(LF/HF), respectively) [38]. The overall mean walking period was used in the analysis.

Systolic blood pressure (SBP), diastolic blood pressure (DBP), and pulse rate in the right upper arm were measured by an oscillometric method using a digital blood pressure monitor (HEM1020, Omron, Kyoto, Japan). Each was measured twice; if the two values of SBP differed by >10 mmHg and/or the DBP values differed by >6 mmHg, an additional measurement was taken. The mean of the two or three measurements was used in the analysis.

### 2.4. Psychological Measurements

The psychological evaluations used the modified semantic differential (SD) method, the Profile of Mood State (POMS) and State–Trait Anxiety Inventory (STAI) questionnaires. The SD method obtains a subjective assessment from the participant through a questionnaire comprising pairs of opposing adjectives, each evaluated on a 13-point scale [39]. Three pairs of adjectives were assessed: “comfortable–uncomfortable”, “relaxed–awakening”, and “natural–artificial”.

The POMS is a well-established measure of psychological distress derived from factor analysis, and its high levels of reliability and validity have been documented [40,41]. It simultaneously evaluates six moods: tension and anxiety (T–A), depression and dejection (D), anger and hostility (A–H), fatigue (F), confusion (C), and vigor (V). The total mood disturbance score was calculated from the formula [(T–A) + (D) + (A–H) + (F) + (C) – (V)] [40]. The total mood disturbance score is understandable from a clinical perspective and intercorrelations among the six primary POMS factors suggest that it is highly reliable [41]. In this study, we used the Japanese version of the POMS in a short form with 30 questions [42] to reduce the burden on the participants.

The STAI form X was used to assess the participants’ state anxiety level. STAI is a self-reported tool that measures the current presence and severity of symptoms of anxiety and a generalized propensity to be anxious [43]. State anxiety is a measure of the current state of anxiety (how the respondent feels “right now”), as assessed by 20 questions [44]. 

### 2.5. Data Analysis

The analysis included the HRV and heart rate data of 52 participants and the blood pressure and pulse rate data of 46 participants, because of errors in the data collection. The statistical analyses were performed using SPSS version 20.0 (IBM Corp., Armonk, NY, USA). Paired t-tests were used to compare physiological responses between the forest and city areas. The Wilcoxon signed-rank test was used to compare the psychological responses. For all the analyses, a *p*-value <0.05 was considered statistically significant. One-sided tests were used because it was hypothesized that the participants would be physiologically and psychologically relaxed by walking in forests.

## 3. Results

**Figure 2:**
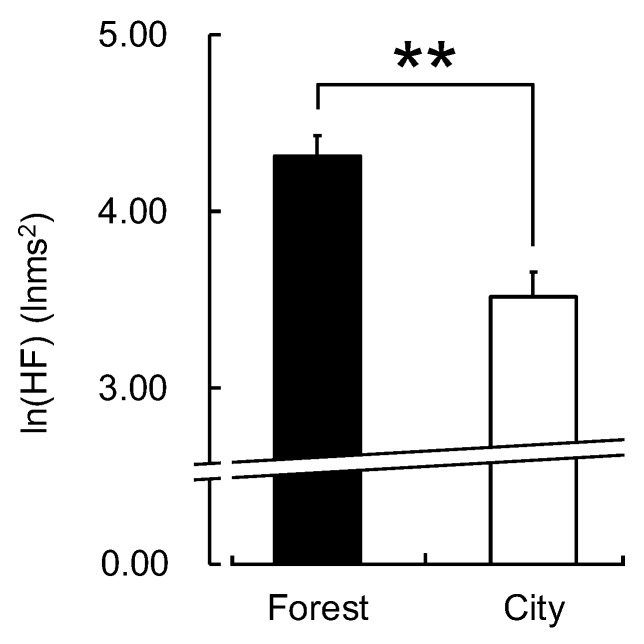
The natural logarithm of the high-frequency power component of heart rate variability while walking in forest and city areas. Data are shown as means ± standard error (n = 52). ** *p* < 0.01, paired *t*-test (one-sided).

**Figure 3:**
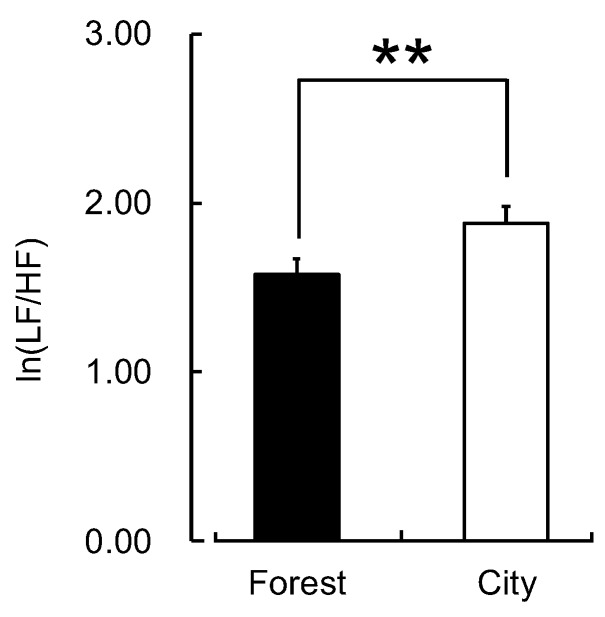
The natural logarithm of the heart rate variability low frequency/high frequency ratio while walking in forest and city areas. Data are presented as means ± standard error (n = 52). ** *p* < 0.01, paired *t*-test (one-sided).

**Figure 4:**
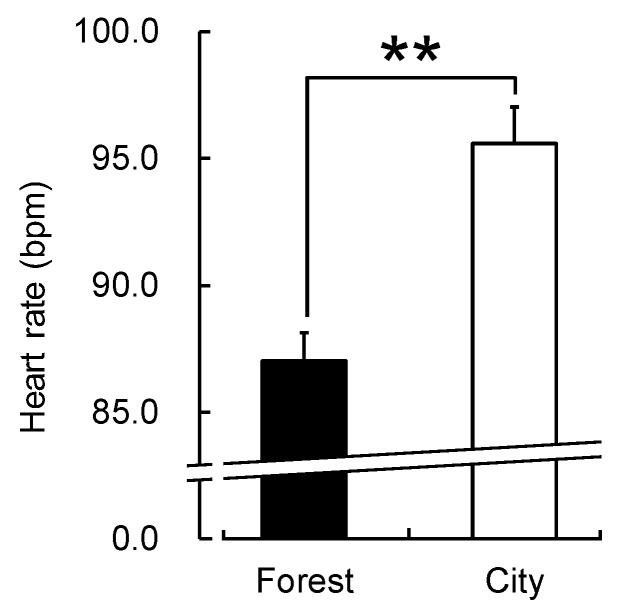
Heart rate during walking in forest and city areas. Data are shown as means ± standard error (n = 52). ** *p* < 0.01, paired *t*-test (one-sided).

There was no significant difference between the two environments in the participants’ walking speed (forest: 3.63 ± 0.62 km/h; city: 3.73 ± 0.74 km/h; *p* > 0.05). However, the participants exhibited statistically significant differences in their physiological and psychological responses to the walks in the forest and city areas. The mean value of ln(HF), an indicator of parasympathetic nervous activity, averaged over the entire walking period, was significantly higher for forest walking than for city walking (forest: 4.31 ± 0.12 lnms^2^; city: 3.52 ± 0.14 lnms^2^; *p* < 0.01; Figure 2). There was also a significant difference in the original, non-logarithmic HF values (forest: 105.12 ± 14.01 ms^2^; city: 57.11 ± 10.36 ms^2^; *p* < 0.01). In contrast, ln(LF/HF), an indicator of sympathetic nervous activity, was significantly lower during forest walking than during city walking (forest: 1.58 ± 0.09; city: 1.88 ± 0.10; *p* < 0.01; Figure 3). There was also a significant difference in the LF/HF ratio values before taking the logarithm (forest: 6.10 ± 0.67; city: 8.19 ± 0.76; *p* < 0.01). In addition, the mean heart rate was significantly lower during forest walking than during city walking (forest: 87.0 ± 1.1 bpm; city: 95.6 ± 1.4 bpm; *p* < 0.01; Figure 4).

There were no significant differences in SBP or DBP between the forest and city areas (SBP after the forest walk: 97.3 ± 1.2 mmHg; SBP after the city walk: 97.5 ± 1.2 mmHg; *p* > 0.05; DBP after the forest walk: 59.8 ± 0.9 mmHg; DBP after the city walk: 59.2 ± 1.1 mmHg; *p* > 0.05). There were no significant differences in the blood pressure values before and after walking in either area. The pulse rate after walking differed significantly between the forest and city areas (forest: 69.3 ± 1.2 bpm; city: 71.9 ± 1.3 bpm; *p* < 0.05), although there was no significant difference before walking (forest: 67.9 ± 1.1 bpm, city: 69.4 ± 1.3 bpm; *p* > 0.05). The pulse rate increased between before and after walking in both areas. 

**Figure 5:**
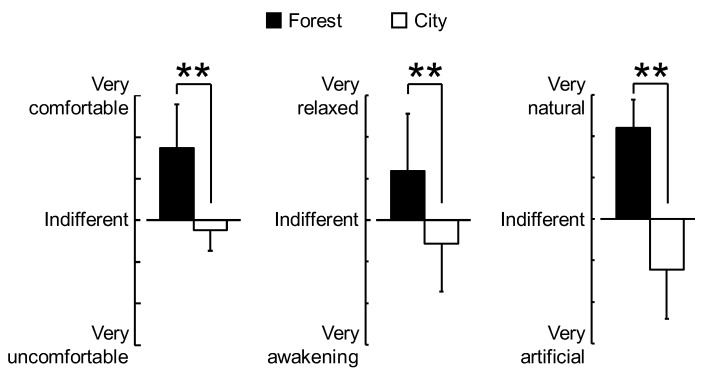
Subjective feelings after walking in forest and city areas, assessed using the modified semantic differential method. Data are shown as means ± standard deviation (n = 60). ** *p* < 0.01, Wilcoxon signed-rank test (one-sided).

Significant differences between the forest and city experiments were observed for all the psychological measures. Figure 5 shows the results of the modified SD method. The subjective evaluations indicated that the participants felt significantly more “comfortable,” “relaxed,” and “natural” when walking in forests than when walking in city areas (all *p* < 0.01).

**Figure 6:**
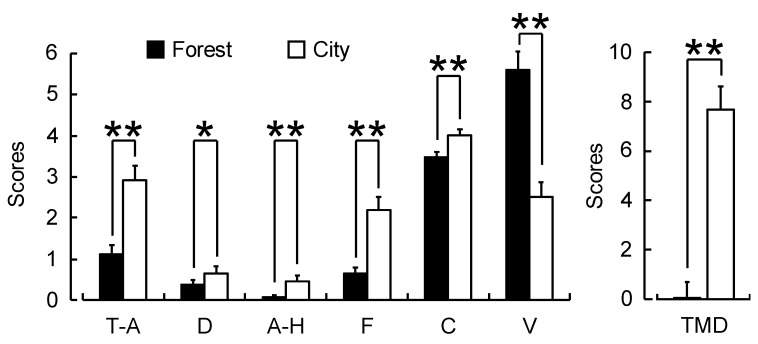
Subjective feelings as measured by the six subscales and the total mood disturbance (TMD) score of the Profile of Mood States questionnaire after walking through forest and city areas. Data are shown as means ± standard deviation (n = 60). ** *p* < 0.01, * *p* < 0.05, Wilcoxon signed-rank test (one-sided). T–A, tension and anxiety; D, depression and dejection; A–H, anger and hostility; F, fatigue; C, confusion; V, vigor.

Significant differences between the forest and city areas were observed for all the POMS subscales rated after walking and for the total mood disturbance score (Figure 6). The subscale scores for the forest and city areas were as follows: tension and anxiety subscale, 1.1 ± 1.8 vs. 2.9 ± 2.8; depression and dejection, 0.4 ± 0.8 vs. 0.6 ± 1.4; anger and hostility 0.1 ± 0.3 vs. 0.5 ± 1.1; fatigue, 0.6 ± 1.1 vs. 2.2 ± 2.5; and confusion, 3.5 ± 1.0 vs. 4.0 ± 1.2. All these negative mood state scores were significantly lower after walking in the forest than after walking in the city (all *p* < 0.01, except for depression and dejection, *p* < 0.05). In contrast, the vigor subscale score after walking in the forest was 5.6 ± 3.5, which was significantly higher than 2.5 ± 2.8 after walking in a city area (*p* < 0.01). The total mood disturbance score was significantly lower after walking in a forest area than after walking in a city area (forest: 0.1 ± 4.9; city: 7.7 ± 7.3; *p* < 0.01).

**Figure 7:**
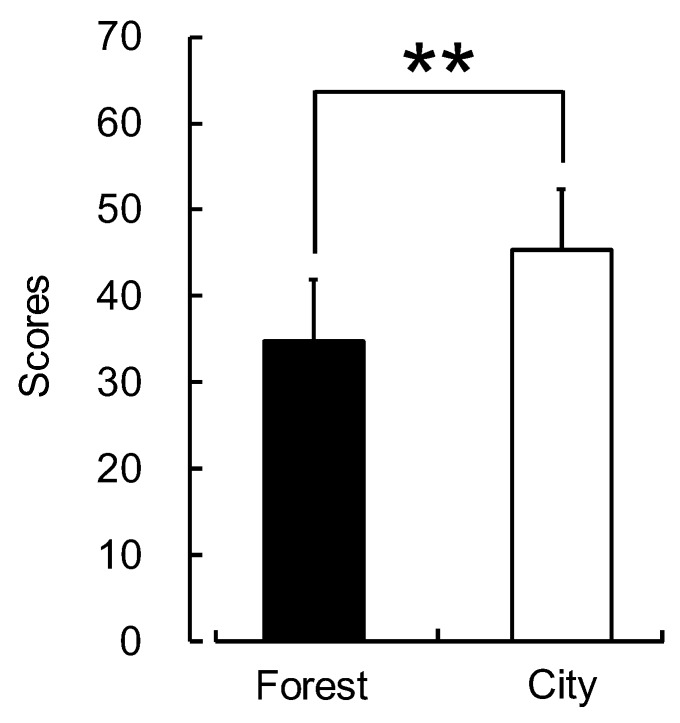
Subjective feelings after walking through forest and city areas as measured by the state anxiety score of the State–Trait Anxiety Inventory questionnaire. Data are expressed as means ± standard deviation (n = 60). ** *p* < 0.01, Wilcoxon signed-rank test (one-sided).

Finally, the state anxiety score of the STAI was 34.8 ± 7.2 after walking in a forest area, significantly lower than 45.3 ± 7.1 after walking in a city area (*p* < 0.01; Figure 7).

## 4. Discussion

The present study assessed the physiological and psychological effects on young women of a brief walk through a forest. 

Compared with walking in a city environment, a brief walk in a forest environment significantly increased the participants’ parasympathetic nervous activity while reducing their sympathetic nervous activity and heart rate. This was consistent with findings of previous studies that investigated physiological responses to a forest environment in men [5,7,9,10,11,12,13,16,17,19,25,28]. The increase in parasympathetic nervous activity and suppression of sympathetic nervous activity suggested that a brief walk in a forest induces physiological relaxation. 

However, there were no significant differences between the forest and city environments in the changes in the participants’ blood pressure. The mean SBP value before walking was 95.6 ± 1.2 mmHg in the forest area and 95.9 ± 1.3 in the city area, and DBP was 58.6 ± 1.0 mmHg in the forest area and 58.8 ± 1.0 in the city area. Blood pressure in the range 120–129/80–85 mmHg is widely regarded as normal, with <120/80 mmHg often cited as the optimal blood pressure [45], although this remains controversial. The mean blood pressures in this study seem to be very low, which meant it was necessary to raise the blood pressure closer to an appropriate level. A previous study found that participants with initially high blood pressure showed a decrease in blood pressure after walking in a forest, whereas those with initially low blood pressure showed an increase, suggesting that a forest environment can be used to help achieve an appropriate blood pressure [46]. From now on, it seems necessary to examine this point.

The psychological evaluations were generally in accordance with the physiological responses. Scores for the comfortable, relaxed, and natural parameters as well as the vigor subscale of the POMS were significantly higher after walking in a forest than after walking in a city area, whereas the scores for the negative feeling subscales, including tension–anxiety, depression–dejection, anger–hostility, fatigue, and confusion, were significantly lower, as was the total mood disturbance score and the STAI anxiety dimension score. These results, which demonstrate the psychological benefits of forests, are partly consistent with previous findings of the effects of viewing forest scenery or walking in forests on men [9,11,47,48]. Recent research has shown that city dwellers are constantly exposed to stressors and that urban living is associated with an increased risk of health problems [49,50,51,52]. In particular, mental health problems can be profound. Current city dwellers have a 39% higher risk for mood disorders and a 21% higher risk for anxiety disorders than those living in rural areas [50], and they have higher rates of psychotropic medication prescriptions for anxiety, depression, and psychosis [52]. Therefore, the psychological benefits of walking through forests could be clinically significant, and it is expected that forest environments will play very important roles in promoting mental health in the future.

The results of this study provide evidence that walking in a forest environment induces physiological and psychological relaxation in young women. However, the study had several limitations. First, the participants were limited to healthy female university students in their 20 s. To generalize the findings, further studies are needed that include other demographic groups, including individuals of different ages. Second, the only physiological indices used were for autonomic nervous activity. For a more complete picture, a comprehensive examination using multiple indices, such as brain and endocrine activity, is necessary. These limitations should be considered in future research.

## 5. Conclusions

Our study revealed the following noteworthy findings regarding the effects of walking in a forest on young women: (1) higher parasympathetic nervous activity; (2) lower sympathetic nervous activity; (3) lower heart rate; (4) feeling more “comfortable”, “relaxed”, and “natural”, as assessed by the modified SD method; (5) more improvement in the mood state, as assessed by the POMS; and (6) lower the anxiety level than those caused by walking in the city area. In conclusion, brief walks in forests induced physiological and psychological relaxation in healthy young women. 

## Figures and Tables

**Table 1:** Participant characteristics (N = 60).

Parameter	Mean ± Standard Deviation
Age (years)	21.0 ± 1.3
Height (cm)	158.4 ± 5.3
Weight (kg)	50.7 ± 6.8
BMI ^1^ (kg/m^2^)	20.2 ± 2.3

^1^ BMI: body mass index.

**Table 2:** The details regarding the experiment sites in the forest.

	1	2	3	4	5	6
**Experimental location**	Iwate Town, Iwate Prefecture	Motosu City, Gifu Prefecture	Shiso City, Hyogo Prefecture	Daigo Town, Ibaraki Prefecture	Hakone Town, Kanagawa Prefecture	Oi Town, Kanagawa Prefecture
**Experimental period**	5–6 August 2014	12–13 August 2014	21–22 August 2014	6–7 August 2015	2–3 September 2015	5–6 September 2017
**Forest physiognomy**	Secondary forest (Red pine & Oak) and artificial forest (Larch)	Secondary forest (Oak & Cherry)	Secondary forest (Oak & Maple)	Secondary forest (Red pine & Oak)	Secondary forest (Red pine & Oak)	Secondary forest (Oak) and artificial planting (Ginkgo)
**Weather**	1st day: sunny	1st day: sunny	1st day: sunny	1st day: sunny	1st day: rainy	1st day: sunny
	2nd day: sunny	2nd day: sunny	2nd day: rainy	2nd day: sunny	2nd day: cloudy	2nd day: cloudy
**Temperature (℃) (mean ± SD ^1^)**	Forest: 26.6 ± 0.5	Forest: 28.3 ± 1.0	Forest: 25.3 ± 1.4	Forest: 33.3 ± 0.8	Forest: 26.3 ± 0.5	Forest: 24.8 ± 0.4
	City: 28.1 ± 0.6	City: 29.1 ± 0.8	City: 29.9 ± 1.2	City: 35.9 ± 0.8	City: 27.7 ± 0.3	City: 27.4 ± 0.6
**Humidity (%) (mean ± SD)**	Forest: 83.5 ± 2.3	Forest: 75.5 ± 5.3	Forest: 83.0 ± 5.0	Forest: 54.2 ± 3.1	Forest: 75.5 ± 1.3	Forest: 77.9 ± 1.9
	City: 74.9 ± 2.9	City: 68.6 ± 3.3	City: 63.6 ± 5.2	City: 50.2 ± 2.2	City: 72.4 ± 2.8	City: 62.7 ± 2.2
**Illuminance (lx) (mean)**	Forest: 2420	Forest: 7390	Forest: 5670	Forest: 1040	Forest: 1110	Forest: 1920
	City: 7280	City: 7630	City: 10,300	City: 9530	City: 3760	City: 4980

^1^ SD: standard deviation.
